# Harnessing genetic diversity: The genomic and transcriptomic insights of *Eugenia uniflora* for environmental resilience

**DOI:** 10.1371/journal.pone.0333437

**Published:** 2025-11-04

**Authors:** Isabel Cristina Cadavid Sánchez, Edgar L. Waschburger, Rita M.C. de Almeida, Alexandre Nascimento de Vargas, Guilherme Loss de Morais, Jimena Giraldo Flores, Dámaris Esquén Bayona, Rogerio Margis, Andreia Carina Turchetto-Zolet, Frank Lino Guzman Escudero

**Affiliations:** 1 Universidade Federal do Rio Grande do Sul, Centro de Biotecnologia, Departamento de Biofísica, Brasil; 2 Universidade Federal do Rio Grande do Sul, Instituto de Biociências, Departamento de Genética, Programa de Pós-Graduação em Genética e Biologia Molecular, Brasil; 3 Instituto de Física, Universidade Federal do Rio Grande do Sul, Porto Alegre, Rio Grande do Sul, Brasil; 4 Instituto Nacional de Ciência e Tecnologia, Sistemas Complexos, Universidade Federal do Rio Grande do Sul, Porto Alegre, Rio Grande do Sul, Brasil; 5 Programa de Pós Graduação em Bioinformática, Universidade Federal do Rio Grande do Norte, Natal, Rio Grande do Norte, Brasil; 6 Biotrop Soluções Biológicas, Curitiba, Brasil; 7 Laboratorio de Biomoléculas, Facultad de Ciencias de la Salud, Universidad Peruana de Ciencias Aplicadas, Lima, Perú; Kerman University of Medical Sciences, IRAN, ISLAMIC REPUBLIC OF

## Abstract

Pitanga (*Eugenia uniflora* L.), a member of the Myrtaceae family, is native to the Brazilian Atlantic Forest and distributed across various ecological environments, including regions with contrasting edaphoclimatic conditions. Known for its production of secondary metabolites with significant biological activity, pitanga holds considerable pharmacological potential. Genomic and transcriptomic resources for this species are therefore valuable for understanding the genetic mechanisms that enable its adaptation to diverse ecosystems and for identifying candidate genes relevant for crop improvement and bioprospection. To explore whether genetic diversity is associated with population adaptation to environmental conditions, we first generated a draft genome of *E. uniflora,* totaling 385.1 Mbp with an N50 value of 26,199 bp, assembled *de novo* from Illumina-sequence reads. Likewise, gene prediction, based on Viridiplantae protein references, identified 30,663 protein-coding genes. Comparative genomics revealed 2,219 orthologous clusters, 40% of which were functionally annotated and encompassing 1,772 gene ontology terms. This draft genome also facilitated the identification of microsatellite markers, whose variation was analyzed across pitanga samples from two contrasting natural environments: Restinga and Riparian forest. The microsatellite profile showed a natural bias towards monomeric repeats, with genetic diversity differences across both populations that could be used as molecular markers for phenotype selection and plant breeding. Furthermore, RNA sequencing coupled with a *Transcriptogram* approach revealed significant differences in gene expression between the two populations. Pitangas from the Restinga ecosystem exhibited a stronger stress response, with distinct gene expression patterns associated with osmoprotection, cell wall modification, detoxification, nutrient balance, and epigenetic regulation. These patterns are likely linked to enhanced adaptation to the water and osmotic stress conditions, characteristic of this environment. Together, these findings enhance our knowledge of genetic diversity within *E. uniflora* populations and the molecular basis of their environmental adaptability. Such insights are critical for understanding how plants rapidly adapt to climate change and how these adaptations affect population dynamics, important for conservation strategies, population management, and the development of resilient cultivars.

## 1. Introduction

*Eugenia uniflora* (Myrtaceae), commonly known as pitanga, is native to the Atlantic Forest and is naturally distributed throughout Brazil [1,2], northern Argentina, and Uruguay. Pitanga produces edible cherry-like fruits and leaves rich in a variety of secondary metabolites, including volatile oils such as terpenes, flavonoids, tannins, and steroids [2], which exhibit extensive pharmacological potential.

This species has a broad ecological range, thriving in various edaphoclimatic conditions. In the Restinga ecosystem (RE) — coastal, dry, and sandy areas characterized by salt- and nutrient-poor conditions — the species typically grows as a shrub. In contrast, it develops into a tree in Riparian Forests (RF) or gallery forests, which are located along freshwater bodies with rich, moist soil [3]. The biological properties of *Eugenia uniflora*, including its adaptability to different habitats and the diversity in its essential oil composition in response to environmental factors, make it a significant model for studying plant adaptive differences and responsiveness to abiotic stressors. Genomic and transcriptomic resources serve as powerful tools for elucidating the molecular mechanisms involved in these adaptations. Genomic sequences for *Eugenia uniflora* are scarce compared to other members of the Myrtaceae family, such as the better-studied *Eucalyptus grandis* [4]. Previous studies sequenced the *pitanga* genome, totaling 3.15 Mb, to identify SSR (Simple Sequence Repeat) markers, investigate repetitive sequences, and predict genes [5]. However, a more comprehensive approach is needed for a deeper and more extensive analysis. Hence, this study aimed to sequence and annotate the nuclear genome of *Eugenia uniflora* to catalog its unique genes. This information was utilized to identify and characterize SSR markers from samples collected across natural habitats, specifically in Restinga (RE) and Riparian Forest (RF). In this study, we also investigate how genetic and transcriptomic diversity among *Eugenia uniflora* populations corresponds to their adaptive strategies in response to contrasting environmental stresses. Specifically, we hypothesize that variations at the molecular level underpin differences in stress tolerance mechanisms that enable survival and growth in the nutrient-poor, saline conditions of the Restinga ecosystem versus the moist, nutrient-rich Riparian Forest.

Research by Turchetto-Zolet et al. (2024) [6] demonstrated that gene expression patterns in *E. uniflora* vary between these environments, highlighting the contrasting adaptations of each ecosystem. Based on this knowledge, we conducted a drought stress experiment to evaluate the differing responses of plants from the two habitats. A Transcriptogram approach was employed for this analysis, similar to previous studies in *Arabidopsis* [7] and soybean [8,9]. This approach uses *top-down* analyses that begin with significantly altered biological functions and then refine the investigation to hierarchically uncover the relevant pathways and genes associated with those functions. Consequently, whole-genome-level expression changes are graphically represented, allowing for the identification of significantly affected pathways [10]. This strategy facilitates comparisons between plants from both habitats and helps pinpoint genes that may play a role in the adaptation of different *pitanga* genotypes.

## 2. Materials and methods

### 2.1. Study permissions and specimen collection, DNA extraction, and sequencing for genome assembly

Specimens used for genome and transcriptome analyses were collected in accordance with research permits issued by Brazilian regulatory institutions: the National System for the Management of Genetic Heritage and Associated Traditional Knowledge (SisGen; Nos. ABE91D8, A7B0331), the Authorization and Information System on Biodiversity (SISBio; No. 74803), and the State Institute of the Environment of Rio de Janeiro (INEA; No. 075/2022).

For genome sequencing, young leaves from a pitanga tree were collected at the Universidade Federal do Rio Grande do Sul (UFRGS), Porto Alegre, Brazil (S: 30°4’2.71“; W: 51°7’11.88”), and deposited in the Herbarium of the Natural Sciences Institute at the same university. Total DNA was extracted using the CTAB method [11], and its quality was assessed by agarose gel electrophoresis and a Nanodrop spectrophotometer (Nanodrop Technologies, USA). A 10 µg sample of DNA was sent to Macrogen (Korea) for sequencing preparation. Paired-end (PE) and mate-paired (MP) libraries were prepared. For the PE library, DNA was fragmented to a maximum size of 500 bp by sonication, and the TruSeq DNA Sample Preparation Kit was used to construct the library with an insert size of 550 bp. For the MP library, DNA was fragmented to a maximum size of 8 Kbp, and the Mate Pair v2 kit was used for library construction, with an insert size of 8 Kbp. Sequencing was performed using the Illumina HiSeq 2000 platform. Sequences are available under Bioproject PRJNA784246. In addition to the libraries produced here, the paired-end library from Eguiluz et al. (2017) [12] obtained from the same tree was used in the genome assembly.

### 2.2 Quality evaluation of sequencing libraries

The quality of the reads and the presence of adapters were evaluated using the FastQC program (www.bioinformatics.babraham.ac.uk/projects/fastqc/). Subsequently, adapter sequences and low-quality reads (Phred score < 30) were removed using NextClip [13] and Trim Galore! [14] for the mate-paired (MP) and paired-end (PE) libraries, respectively. The cleaned reads were mapped onto the pitanga chloroplast genome [12] (Eguiluz et al. 2017) using Bowtie 2 [15], and these reads were subsequently excluded.

### 2.3. Genome size estimation and de novo assembly

Filtered paired-end libraries were used for k-mer counting with Jellyfish [16], using a k-mer size of 21. The distribution of k-mer counts was visualized in GenomeScope v1.0.0 [17] to estimate genome size, heterozygosity, and the proportions of unique and repetitive sequences. The pitanga *de novo* genome was assembled using the Platanus program [18] with default parameters. Initially, Platanus assembled contigs from paired-end library reads by extending k-mers to construct De Bruijn graphs. Contig ordering was then determined to generate scaffolds using information from both paired-end and mate-pair (MP) libraries, with insert sizes estimated based on read pairs mapping to the same contig. Finally, the remaining gaps were closed using reads mapped to the scaffolds. The quality of the assembly was assessed using the QUAST program [19], which considers a minimum scaffold size of 800 bp.

### 2.4. Identification and classification of repetitive sequences in the pitanga genome

To identify repetitive sequences in the genome before predicting protein-coding genes, we first used the RepeatModeler program (https://github.com/rmhubley/RepeatModeler) with default parameters. This program performs a BLAST search to detect repetitive elements. Next, the RepeatScout program (http://www.repeatmasker.org/) was employed to construct a repeat library based on the results from RepeatModeler. This preliminary library was then used with RepeatMasker (http://www.repeatmasker.org/) to perform an initial search of the genome and remove repetitive sequences that occur at least ten times. To ensure that conserved protein families were not mistakenly identified as repeats, we used the BLAST2GO program [20] to compare the putative repeats against known proteins via BLAST. Additionally, the TEclass program [21] was used to classify the repeats into four categories based on their transposition mechanisms: DNA transposons, LTRs (long terminal repeats), LINEs (long interspersed nuclear elements), and SINEs (short interspersed nuclear elements). Finally, using the filtered and classified repeat library, we performed soft masking of the pitanga genome with RepeatMasker.

### 2.5. Gene prediction and annotation refinement in genome assembly

For gene prediction, we used the BRAKER2 pipeline [22], which provides a more accurate and comprehensive annotation strategy compared to traditional *ab initio* approaches by combining transcriptomic evidence and protein homology. This pipeline automates the training of Augustus [23] and GeneMark-ES/ET/EP [24] using protein homology and RNA-seq data. Augustus was used for *ab initio* predictions, while GeneMark-ES/ET/EP benefited from homology-based cues provided by the ProtHint program [25] and the alignment of RNA-seq reads. ProtHint maps predicted genes to the OrthoDB database (https://v100.orthodb.org/download/odb10_plants_fasta.tar.gz) and protein sequences from the *Eucalyptus grandis* genome (https://phytozome-next.jgi.doe.gov/), offering information such as introns, start codons, and stop codons. Simultaneously, reads from the RNA-seq libraries (described in the following section) were aligned onto the masked genome using HISAT2 [26]. Augustus was trained using both hints and applied to gene prediction. The BRAKER2-generated annotation file was then refined by removing coding sequences lacking start and stop codons, as well as overlapping exons, using the gFACs program [27], which aids in filtering and analyzing genome annotations. Genes overlapping masked regions by 95% or more were excluded using in-house scripts and the PRINSEQ program [28], which provides tools for filtering and summarizing sequence data. Finally, the assembly quality and gene prediction were validated with the BUSCO program [29], using the eudicots_odb10 database to assess completeness based on single-copy orthologs.

### 2.6. Comparative genomics analysis

We used OrthoVenn2 [30] with an inflation value of 1.5 to generate ortholog clusters from the predicted proteins of the *Eugenia uniflora* genome. For comparison, we included protein sequences from the genomes of *Arabidopsis thaliana*, *Eucalyptus grandis*, *Populus trichocarpa*, and *Vitis vinifera*, all sourced from the Phytozome database [31]. All proteins were compared for orthology using all-against-all alignments with an e-value threshold of 10^-5. As described previously, proteins unique to pitanga-specific ortholog clusters were functionally annotated using the BLAST2GO program.

### 2.7. Transcriptomic analysis and differentially expressed genes under drought stress

Details of plant material collection in natural environments and growth and drought stress treatments are provided by Anton et al. (2020) [32]. Briefly, seeds to produce experimental plants were sourced from two distinct environments in Brazil: Restinga and Riparian Forest. Three-month-old *Eugenia uniflora* plants, cultivated in a greenhouse, were subjected to drought stress by halting irrigation for 14 days. Physiological assessments confirmed the impact of the stress treatment on plant biomass and photosynthesis, showing a substantial reduction in plant weight and stomatal conductance in both populations compared to the controls [32]. Leaf tissue was collected from three plants under control conditions and three plants under drought stress from each population, resulting in a total of 12 RNA-seq libraries. The transcriptomic data have been submitted to the NCBI Sequence Read Archive under Bioproject PRJNA784246. RNA-seq libraries were generated and submitted for quality analysis and trimming as described previously. Cleaned reads were mapped to the *E. uniflora* genome using HISAT2 [26] with default parameters. Read frequencies for each gene were calculated using the featureCounts function of Subread [33]. Statistical analysis was performed using the DESeq2 package [34] implemented in R. Genes with an absolute fold change ≥ 1.5 and a p-value < 0.05 were considered differentially expressed genes (DEGs). Functional annotation of these genes was performed using eggNOG-mapper [35]. Venn diagrams were constructed to identify unique and shared genes using the Interactive Venn tool (https://www.interactivenn.net/).

### 2.8. Transcriptogram construction and statistical analysis under drought stress

Traditional methods often assess gene expression changes gene-by-gene, which can be noisy and disconnected from biological meaning. Transcriptograms, in contrast, order genes by functional association (such as protein-protein interaction networks) and analyze expression changes across sliding windows of functionally related genes. Individual gene expression can fluctuate due to technical or biological variability. By averaging expression over functionally related gene clusters, Transcriptograms reduce random noise and increase the signal-to-noise ratio. Traditional approaches may focus only on significantly differentially expressed genes, potentially missing broader patterns. Transcriptograms assess expression across the entire genome, providing a global view of how gene networks respond over time or conditions [10].

This analysis generates an ordered gene list for *Eugenia uniflora* by filtering gene products that share at least one association as inferred from the STRING Protein-Protein Interaction database with a threshold of confidence scores > 800. Using the Transcriptogramer program (v3.2) (https://www.bioconductor.org/packages/release/bioc/html/transcriptogramer.html), genes are clustered in a way that the distance between them reflects the likelihood of their association. Term enrichment analysis was performed using Transcriptogramer with the clustering data and a Gene Ontology file. The outputs were used to generate a graph representing the density of genes associated with specific biological processes at each position. Peaks in these profiles indicate clusters of genes belonging to particular Gene Ontology (GO) terms, averaged over windows of 61 genes. Their biological function was defined in the STRING database. To visualize gene expression profiles under drought stress, *transcriptograms* were generated. Transcriptograms are the projection of gene expression data onto the ordering, followed by a window average, which was taken here over 61 genes. We obtained 4 average transcriptograms for plants from Restinga or Riparian forest, in control or drought conditions. We then compared the transcriptograms using the control condition as a reference. The input files included an RNA-Seq expression file (read counts), an ordered gene list, and a window radius of 30 genes. The resulting Transcriptogram file averaged gene expression over a 30-gene window centered on each gene, encompassing 30 genes to the left and 30 genes to the right. We chose a radius of 30 based on the analyses presented by da Silva and collaborators [36]. Averaging over these intervals for each gene in the list produces a smoothed mean expression profile. Using the “statistics” tool, average expression values were calculated for each library replicate. Relative expression averages for each gene were computed with the control as the baseline. A graph was plotted in Origin (https://www.originlab.com/) based on gene position and relative expression values. To better explore the specific response of each genotype, differentially expressed genes were selected, using as criteria that they were identified by transcriptogram (R30 p-value <0.01, R0 p-value <0.05), and by DEseq2 analysis (p-value <0.05).

### 2.9. SSR annotation and GO overrepresentation analysis

The genomic sequence of *E. uniflora* and the eighteen available transcriptomes obtained in previous studies [6,32] were annotated for SSRs using the MISA pearl script [37]. Those identified in the transcriptomes were considered transcribed, while the others were not. SSRs within a 100 bp region and with the same nucleotide sequence were considered as one locus for the purposes of interpreting the population diversity analysis. Lastly, annotated *E. uniflora* genes with present SSRs 2 kbp up or downstream were used as a query against the proteome of *A. thaliana* by Blastp [38]. Only the single top hit for each gene was considered, and hits yielding e-values below 1e-20 were discarded. The resulting list of *A. thaliana* genes was then filtered for duplicates and submitted to the PANTHER database [39] for GO overrepresentation analysis. To identify SSRs possibly linked to differential gene expression profiles, the gene count data for each repeat was separated into two groups based on the presence or absence of SSRs. A likelihood ratio test was performed between the null and alternative fitted negative binomial distributions. The resulting p-values were adjusted with the Benjamin-Hochberg procedure.

### 2.10. Inclusivity in global research

Additional information regarding the ethical, cultural, and scientific considerations specific to inclusivity in global research is included in the Supporting Information (S1 File)

## 3. Results

### 3.1. Genome characterization and repetitive sequence identification

**Fig 1:**
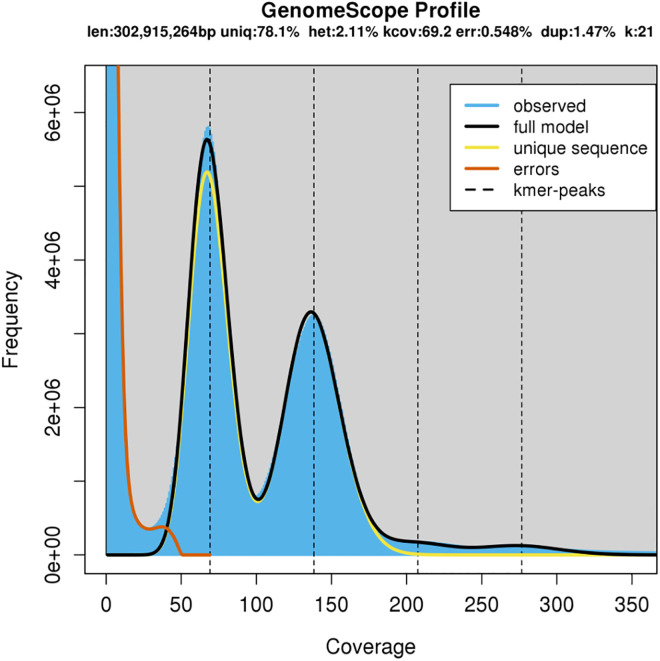
Estimation of the *Eugenia uniflora* genome size. The first peak, located at a coverage of 32X, corresponds to the heterozygous peak, while the second peak at 63X corresponds to the homozygous peak.

After filtering, the sequence depths for the PE 250, PE 550 and MP libraries were 79,984,528, 169,757,171 and 45,718,168, respectively (Table 1). The estimated haploid genome size of *E. uniflora* was 302.9 Mbp with a model adjustment of 97.96% based on k-mer frequency analysis (k = 21). The genome displayed a heterozygosity rate of 2.11%, and 21.9% of its content was identified as repetitive (Fig 1).

**Table 1:** Sequence filtering results.

Library ID	Raw sequences	Filtered sequences	Percentage
PE-250 bp	94140895	79984528	84.96
PE-550 bp	182465435	169757171	93.04
MP	113599977	45718168	40.24

The assembled genome of *E. uniflora* has a total size of 385.1 Mbp (Table 2), distributed across 34,716 scaffolds, with the largest scaffold measuring 361,660 bp. Quality metrics for the assembly are presented in Table 2. Initially, 9,888 repetitive sequences, each occurring more than 10 times in the assembled genome of *E. uniflora*, were identified. Of these, 181 repetitive sequences were excluded due to similarity with protein-coding sequences from Viridiplantae. Using the final set of repetitive sequences, we determined that 194.4 Mbp (50.47%) of the genome is composed of repetitive DNA. Table 3 provides a classification of the identified and masked repetitive sequences in the *E. uniflora* genome.

**Table 2:** Genome assembly statistics.

Metric	Pitanga genome
Total size	385104457
Scaffolds number	34716
Scaffolds >= 5000 bp	16582
Scaffolds >= 10000 bp	10974
Scaffolds >= 25000 bp	4360
Scaffolds >= 50000 bp	1224
Biggest Scaffold	361660
GC (%)	39.81
N50	26199
N75	12057
L50	4080
L75	9489
N’s number per 100 Kbp	568.72

**Table 3:** Classification of the repetitive sequences identified in the *E. uniflora* genome.

Repetition type	Elements #	Occupied size in the genome	%
SINEs	16 586	1 814 643	0.47
LINEs	117 561	23 436 901	06.09
LTR elements	124 214	43 706 929	11.35
DNA elements	313 852	56 620 676	14.70
Unclassified	181 781	68 791 662	17.86

### 3.2. Genome annotation and validation

**Fig 2:**
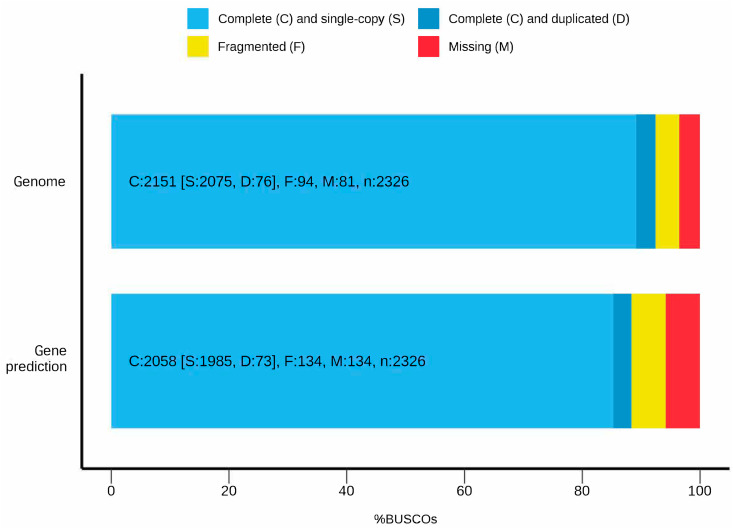
BUSCO validation result comparison for the *Eugenia uniflora* assembled genome and gene prediction.

A total of 32,201 protein-coding genes were predicted in the *E. uniflora* genome. Of these, 95 genes were filtered out because 95% or more of their sequence overlapped with masked regions of the genome. Additionally, 414 genes were excluded for lacking a start codon, 245 for lacking a stop codon, and 784 for having exons overlapping with other genes. The final set of unique protein-coding genes in the *E. uniflora* genome comprises 30,663 genes. Quality validation of the assembled genome using eudicots_odb10 database as a reference revealed that 2,151 genes (92.5%) were complete, while 94 genes (4%) were partial (Fig 2). Validation of the predicted genes yielded 2,058 complete genes (88.4%) and 134 partial genes (5.8%).

### 3.3. Comparative genome analysis and functional annotation of the E. uniflora genome

**Fig 3:**
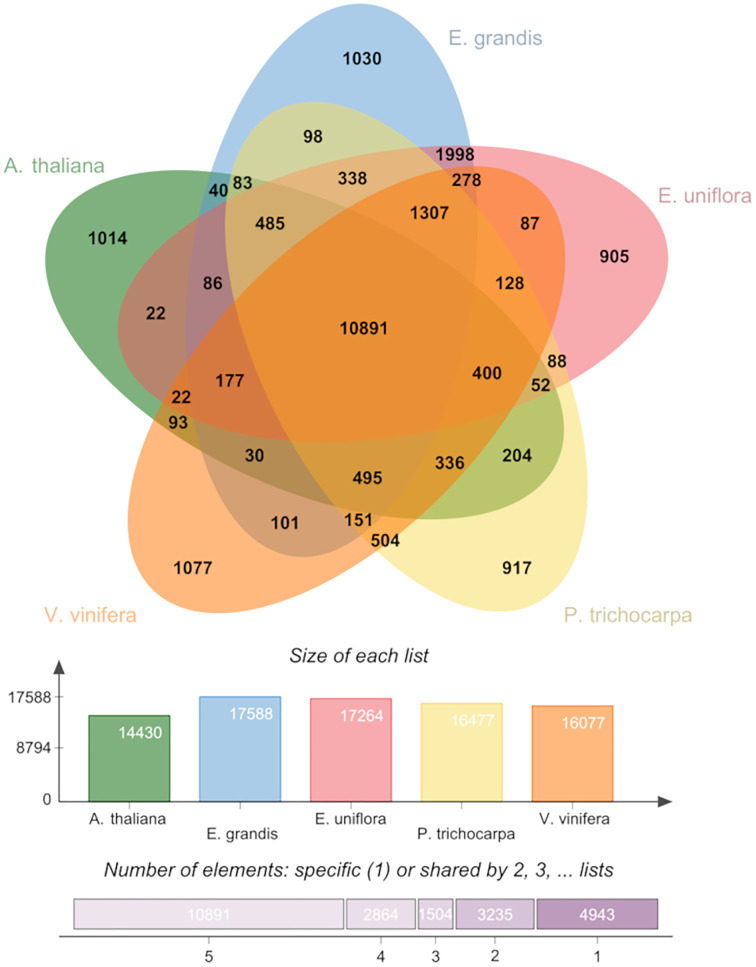
Venn diagram of the orthologous gene clusters identified among the plant species.

**Fig 4:**
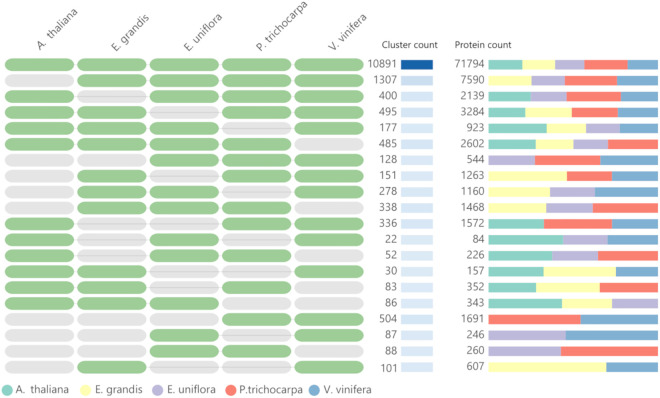
Orthologous clusters and the number of protein-coding genes shared among the plant species.

A total of 30,663 predicted genes in the *E. uniflora* genome were grouped into 17,264 clusters and 9,787 singletons. Table 4 summarizes the clusters and singletons identified in the genomes of *A. thaliana*, *E. grandis*, *P. trichocarpa*, and *V. vinifera*. Comparative analysis revealed 10,891 orthologous clusters shared by all five species (Fig 3). Additionally, 1,030 clusters, comprising 2,219 genes, were identified as specific to *E. uniflora*. Conversely, 1,014 clusters (specific to *A. thaliana*), 1,030 clusters (specific to *E. grandis*), 917 clusters (specific to *P. trichocarpa*), and 1,077 clusters (specific to *V. vinifera*) were also identified. The cluster and gene distribution across the interspecies comparisons are shown (Fig 4). In the comparison between *E. uniflora* and *E. grandis*, 15,560 orthologous clusters were found to be common to both Myrtaceae species (S1 Fig). Additionally, 1,704 clusters were specific to *E. uniflora*, and 1,382 clusters were specific to *E. grandis*.

**Table 4:** Clusters and singletons identified in the plant species of the study.

Species	Proteins	Clusters	Singletons
*Eugenia uniflora*	30663	17264	9787
*Arabidopsis thaliana*	27416	14430	5173
*Eucalyptus grandis*	34349	17588	9305
*Populus trichocarpa*	34699	16477	4957
*Vitis vinifera*	31845	16077	9460

**Fig 5:**
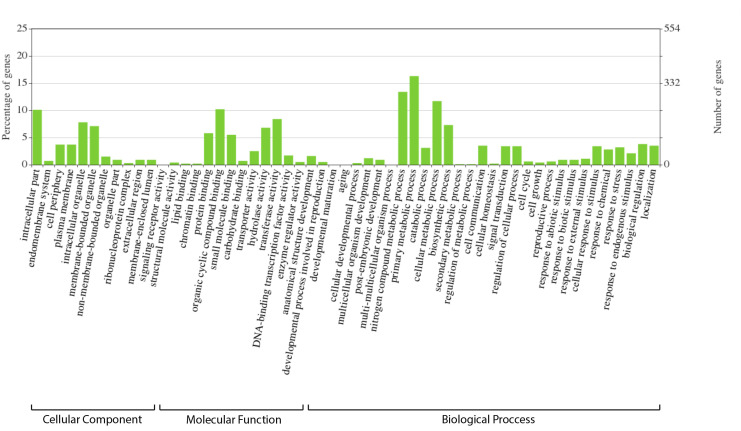
GO categories and terms identified in the *E. uniflora* specific genes.

Annotation of the 2,219 *E. uniflora*-specific genes from the comparative genomic analysis was conducted using data from other Viridiplantae species. Of these, 1,512 genes (68.12%) showed similarity to genes previously reported in different plant species (S1 Table). At the gene ontology (GO) level, 888 genes (40.02%) were functionally annotated, corresponding to 1,772 ontological terms divided into three categories: cellular component, molecular function, and biological process (Table 5). Within the cellular component category, the most abundant terms were “intracellular part” (GO:0044424), “intracellular organelle” (GO:0043229), and “membrane-bounded organelle” (GO:0043227), with 224, 172, and 157 genes, respectively (Fig 5). In the molecular function category, the most common terms were “cyclic organic compound binding” (GO:0097159), “transferase activity” (GO:0016740), and “hydrolase activity” (GO:0016787), with 227, 186, and 150 genes, respectively. In the biological process category, the most frequent terms were “primary metabolic process” (GO:0044238), “nitrogen compound metabolic process” (GO:0006807), and “cellular metabolic process” (GO:0044237), with 361, 298, and 259 genes, respectively. Of particular note, given *E. uniflora*’s potential as a biological model for adapting to diverse environments, terms related to stress and stimulus responses were identified. These included “cellular response to stimulus” (GO:0051716, 75 genes), “stress response” (GO:0006950, 70 genes), “response to chemicals” (GO:0042221, 62 genes), “response to external stimuli” (GO:0009605, 24 genes), and “response to abiotic stimuli” (GO:0009628, 19 genes) (S2 Table).

**Table 5:** GO categories of specific *Eugenia uniflora* genes.

Category	GO terms number
Cellular component	460
molecular function	723
Biological process	589
Total	1772

At the Clusters of Orthologous Genes (COG) level, 609 (60.6%) out of 1005 genes were functionally annotated, corresponding to each category, denoted by a specific letter code. The most abundant category was “Signal transduction mechanisms (T)”, followed by “Posttranslational modification, protein turnover, chaperones (O)” and “Transcription” with 101, 86 and 84 genes, respectively (S2A Fig). The predominance of these categories highlights the importance of regulatory functions, such as adaptability to different environments, defense against pathogens, and stress response, critical for cellular homeostasis. Notably, the category “Carbohydrate transport and metabolism (K)” with 65 genes (6.47%) was also well represented, highlighting the importance of energy metabolism under diverse environmental conditions, playing a crucial role in growth and development.

KEGG pathways related to plant functions were also identified in 273 (27.16%) out of 1005 genes. The most common pathways were “Biosynthesis of secondary metabolites”, “Plant Hormone signal transduction” and “NOD-like receptor signaling pathway” with 140, 38 and 34 genes, respectively (S2B Fig). These findings are consistent with a wide range of metabolites with potential health benefits present in *E. uniflora*, including antioxidants, antimicrobials and anti-inflammatory properties. Moreover, the “Plant Hormone signal transduction” pathway indicates active regulation of development and stress response, which can suggest its adaptation to different environments. The detection of genes involved in “NOD-like receptor signaling pathway”, usually related to immune responses, can reflect defense mechanisms to pathogen attack or stress.

**Fig 6:**
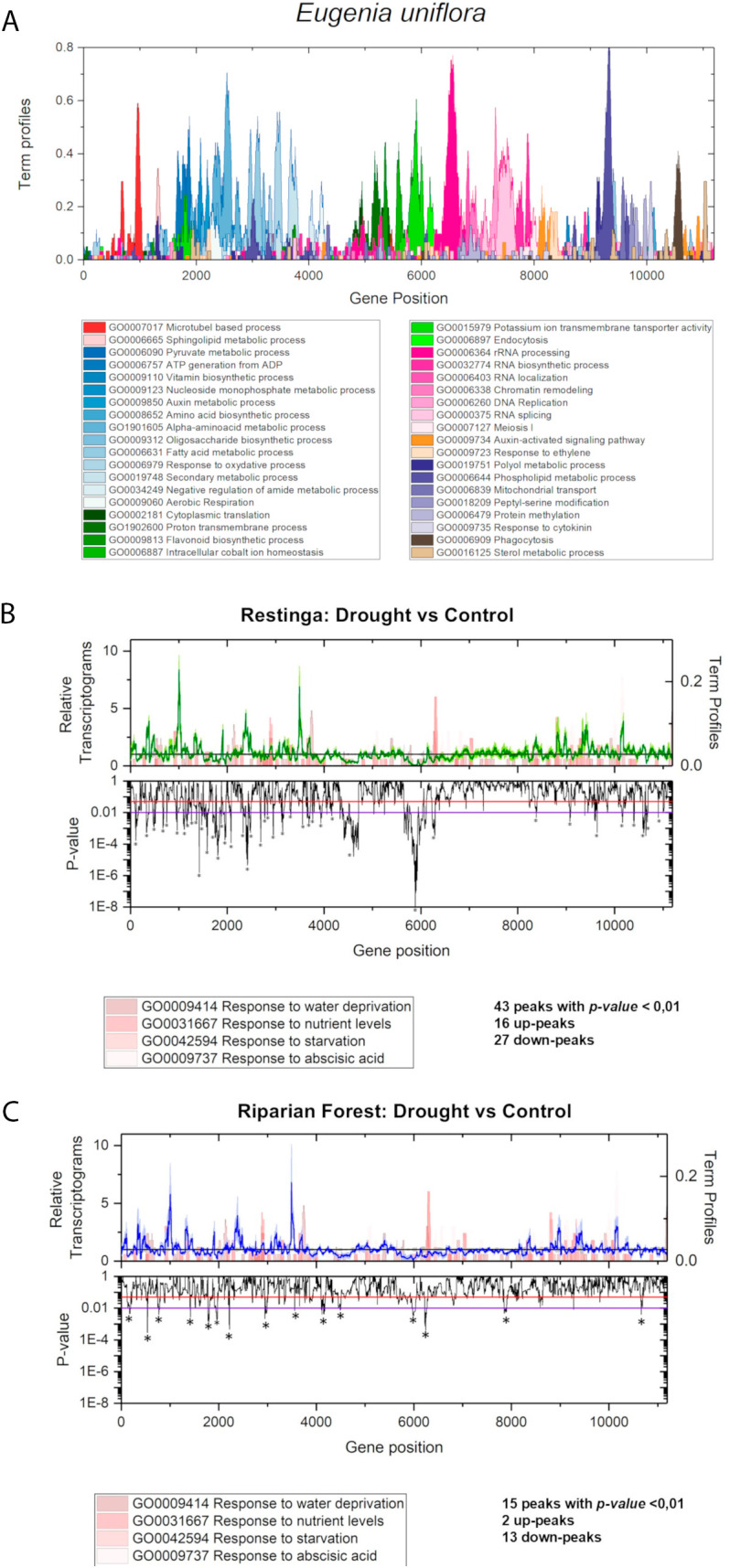
The *transcriptogram* from *Eugenia uniflora.* **(A)** Ordering of *Eugenia uniflora* genes. Closer genes have a higher probability of being associated based on String database information. **(B)** The Restinga transcriptome profile under drought stress. Relative expression data of each gene is plotted. Upper graph: Fold change values are represented on the y-axis, with gene position on the x-axis. Green peaks indicate drought stress samples (deviation is indicated in light-green), while horizontal black lines represent control conditions. GO terms associated with the ordered genes are highlighted in pink tones. Bottom graph: p-values are shown on the y-axis, with gene position on the x-axis. The red line highlights peaks with a p-value < 0.05, while the blue line indicates peaks with a p-value < 0.01 **(C).** The Riparian forest transcriptome profile under drought stress. Relative expression data of each gene is plotted. Upper graph: Fold change values are represented on the y-axis, with gene position on the x-axis. Blue peaks indicate drought stress samples (deviation is indicated in light-blue), while horizontal black lines represent control conditions. GO terms associated with the ordered genes are highlighted in pink tones. Bottom graph: p-values are shown on the y-axis, with gene position on the x-axis. The red line highlights peaks with a p-value < 0.05, while the blue line indicates peaks with a p-value < 0.01.

Carbohydrate-Active enZYmes (CAZy) were identified in 19 genes (1.89%) out of 1005 predicted genes (S2C Fig). The most represented CAZy family were the “GlycosylTransferase Family 1”, followed by “GlycosylTransferase Family 8” with 6 and 3 genes, respectively. Glycosyltransferases are enzymes responsible for the catalysis of glycosidic bonds from phospho-activated sugar donors. “GlycosylTransferase Family 1” (GT1) enzymes usually use UDP-glucose as donors and are involved in the glycosylation of small molecules, including secondary metabolites, affecting the physicochemical and biological activity of these molecules, while also enhancing their stability. In contrast, “GlycosylTransferase Family 8” (GT8) are involved in polysaccharide biosynthesis, with enzymes such as galacturonosyltransferase (GAUT), crucial for the synthesis of homogalacturonan, important component in plant cell wall synthesis. The presence of these GT families highlights the capacity of Eugenia uniflora to engage in a complex carbohydrate metabolism, related to structural maintenance and bioactive compounds modifications.

### 3.4. Differential gene expression in E. uniflora genotypes under drought stress

*Eugenia uniflora* gene ordering was generated using *Transcriptogram*, based on interaction information, including 11,194 interacting genes (Fig 6A). Biological processes for these genes were identified and represented in this graph. The ordered list, available as supplementary material (S3 Table), may be used to produce other *transcriptograms* and analyze data from other *E. uniflora* transcriptome experiments.

The DESeq2 RNA-Seq analysis from the drought stress experiment identified a total of 11,138 differentially expressed genes (DEGs) in pitangas from Restinga (RE), with 5,906 genes up-regulated (141 *E. uniflora*-specific genes) and 5,232 down-regulated (100 *E. uniflora*-specific genes) (S4 Table) when control and stress conditions were compared. In contrast, 7,307 DEGs were detected in pitangas from the Riparian Forest (RF), comprising 4,657 up-regulated (122 *E. uniflora*-specific genes) and 2,650 down-regulated genes (47 *E. uniflora*-specific genes) (S5 Table, an absolute fold change ≥ 1.5 and *p-value* < 0.05). Interestingly, 34.4% of the DEGs in RE were uniquely regulated in this genotype and not in RF plants. These results suggest that RE plants may have a greater responsiveness to drought stress conditions. This observation is further illustrated in the *transcriptograms* (Fig 6B and 6C), which reveal 43 peaks in RE plants compared to 13 peaks in RF plants. These peaks indicate that clusters of genes associated with specific biological processes tend to respond more actively to stress stimuli (R30, p < 0.01).

**Fig 7:**
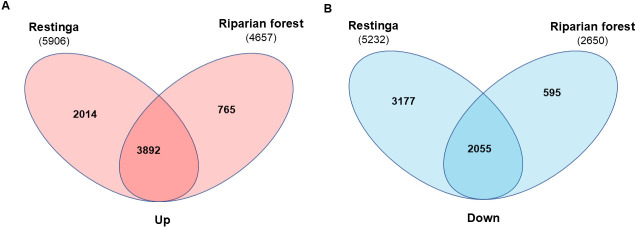
Venn diagram of shared and unique differentially expressed genes between Restinga and Riparian forest under drought stress. Genes that are (A) upregulated are represented in pink, while (B) downregulated genes are shown in blue.

**Fig 8:**
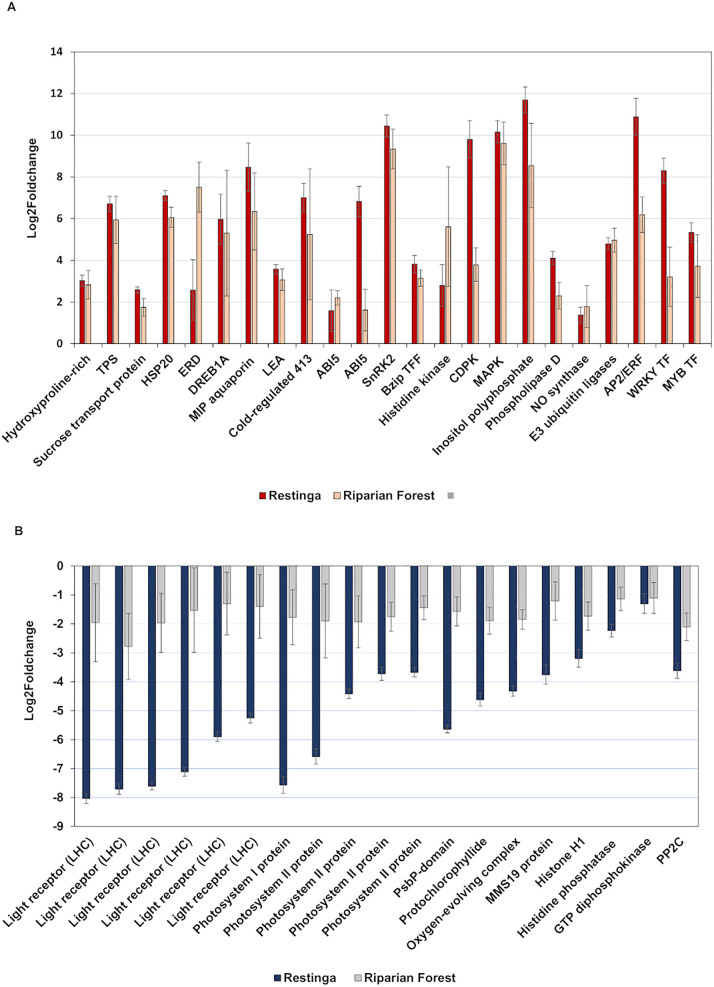
Shared differentially expressed genes between Restinga and Riparian Forest under drought stress. (A) upregulated genes **(B)**. down-regulated genes.

A Venn diagram was used to illustrate the unique and shared genes responsive to stress in RE and RF plants (Fig 7). Among shared genes, 3,892 genes were up-regulated and 2,055 were down-regulated. Gene ontology analysis revealed that both genotypes exhibited typical responses to water deprivation, including the regulation of genes from the abscisic acid (ABA) signaling pathway. Key regulated genes that were identified included the abscisic acid-insensitive 5-like protein (ABI5), protein phosphatase 2C (PP2C), SNF1-related protein kinase (SnRK2), and AREB/ABF-type bZIP transcription factors (Fig 8A). Additionally, components of the ABA-independent pathway, such as dehydration-responsive element-binding proteins (DREBs), were also activated. Other stress response mechanisms observed in both genotypes included osmoprotection through the expression of late embryogenesis abundant (LEA) proteins and trehalose synthases (TPS), as well as cytoskeletal rearrangements, cell wall remodeling (Hydroxyproline-rich glycoprotein), and protein folding (Heat Shock Protein, HSP) and proteolysis (E3 ubiquitin ligases). Second messenger signaling involving calcium, phospholipids, and nitric oxide was also activated. Furthermore, signaling pathways mediated by mitogen-activated protein kinases (MAPKs), WRKY transcription factors, bHLH-MYC, and R2R3-MYB transcription factors were engaged (Fig 8A). Notably, some repressed genes were shared by both genotypes, with a more pronounced response observed in Restinga, particularly those associated with photosynthetic machinery and growth hormones (Fig 8B).

**Fig 9:**
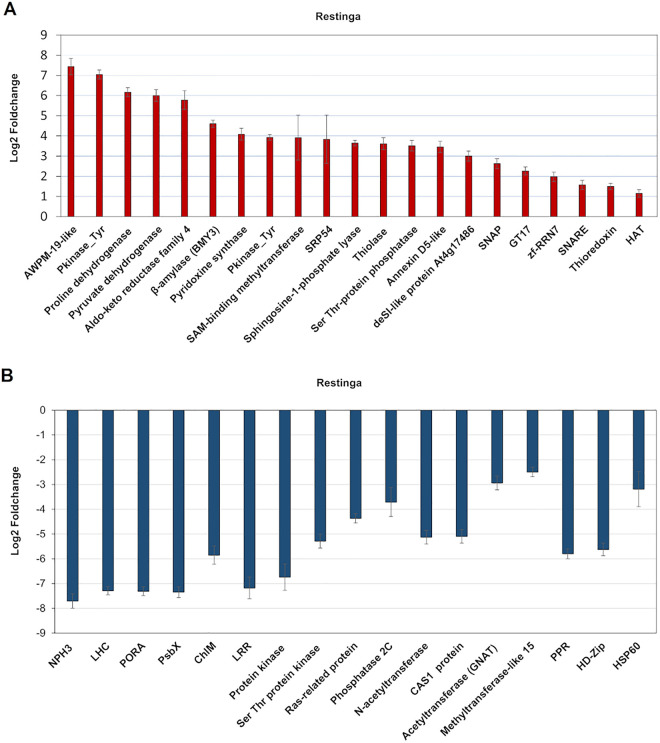
Unique differentially expressed genes of Restinga under drought stress. **(A)** Unique upregulated genes **(B)** Unique down-regulated genes.

This data also reveals that Restinga has unique genes vital for maintaining homeostasis under water deprivation, likely contributing to its plasticity. Notable findings include regulation of transcription, redox homeostasis, detoxification processes, osmotic balance, cell wall modification, nutrient balance, and protein folding (Fig 9). Importantly, potential epigenetic mechanisms, including histone acetylation and methylation, as well as DNA methylation, may facilitate adaptation to conditions distinct from their natural environment.

### 3.5. SSR genomic landscape and population profiles

**Fig 10:**
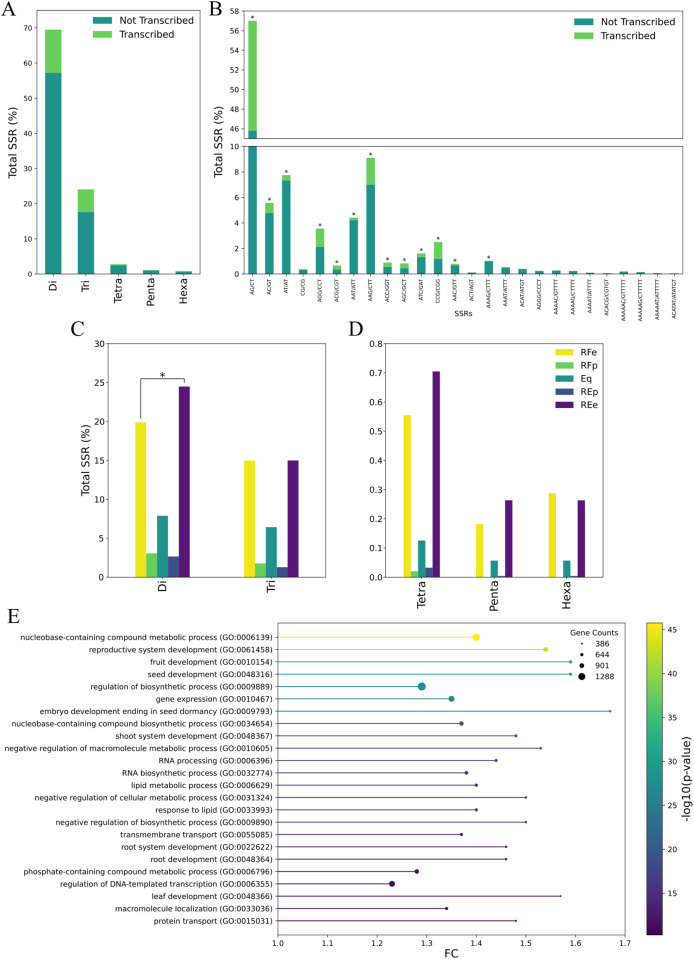
*Eugenia uniflora* SSR metrics. (A) Percentages of SSRs on transcribed and non-transcribed regions grouped by length and (B) sequence. (C) SSR diversity between RE and RF populations. SSR diversity (polymorphisms) is distributed among Riparian Forest (RF) and Restinga (RE) populations in an exclusive (e), equal (eq), or preferential (p) manner. (D) GO overrepresentation analysis on pitanga genes within 2kbp of SSRs. FC values are in log base 2. * denotes 0.01 confidence.

A total of 55,019 SSRs were identified, including both simple and compound formations, representing an SSR density of 142.87 (u/Mbp) (Table 6). They are subdivided into roughly 70% dinucleotide repeats, 25% trinucleotide repeats, and the remaining 5% into tetra- penta- and hexanucleotides (Fig 10A). Nearly a fifth of the dinucleotide SSRs identified were found to be transcribed, representing over 10% of total SSR numbers. When looking at the different possible SSRs within each subdivision, dinucleotides have a clear bias towards the formation of AG/CT repeats, adding to almost 60% of the total SSRs identified (Fig 10B). AG/CT repeats also contribute the most to the number of transcribed SSRs. Interestingly, less than 1% of dinucleotide repeats were GC/GC repeats. Furthermore, chi-square tests were performed to identify which repetitions deviate from the overall SSR proportions (transcribed and not transcribed) for each division (Fig 10B). Every single SSR repetition with over 0.5% representation resulted in a statistically significant deviation. This result indicates that their contributions to transcribed and non-transcribed genomic regions are highly biased regarding sequence content, with AG/CT and AAG/CTT sequences representing a larger share, while CG/CG and ACT/AGT are less preferred.

**Table 6:** SSR identification metrics.

Item	Count
Total number of contigs	34716
Total size of genome (Mb)	385
Total number of identified SSRs	55019
Number of SSR-containing sequences	11339
Number of sequences containing more than 1 SSR	6221
Number of SSRs in compound formation	932
SSR density (u/Mb)	142,87
Di	38239
Tri	13243
Tetra	1533
Penta	607
Hexa	465

* Simple monomeric repeats are not included

When comparing transcribed SSRs between the *E. uniflora* populations of RF and RE (Fig 10C and 10D), we can identify a tendency of RE individuals to present more exclusive SSRs than RF individuals, especially for dinucleotide repeats (chi-square test p-value < 0.01). Although a considerable number of SSRs are shared between RF and RE populations, the majority of their polymorphisms were found to be equally present in both regions, with only a smaller fraction diversified in either one. Moreover, calculated likelihood ratios between gene expression counts for every expressed SSR allowed the identification of SSRs linked to differential gene expression (S6 Table). From the list of DEGs identified under drought conditions, 34 shared responsive genes from the two populations exhibited SSR motifs. Among the unique genes of each population, RE exhibited 16 DEGs with SSRs, while RF exhibited 9 (S7 Table). These results suggest an association between SSR presence and gene expression.

Lastly, a GO overrepresentation analysis was performed on genes within a 2-kbp region of SSR sequences identified across the *E. uniflora* genome. The resulting list of GO biological processes included both broad and specific terms (Fig 10E). In general, SSR proximal genes seem to mostly influence developmental processes in *E. uniflora*. Identified terms encompassed the majority of plant tissues, including leaf, root, root system, shoot system, seed, fruit, and reproductive system development, ordered in increasing p-value. This result is very interesting, given both the ability to use SSRs as molecular markers for phenotype selection and their apparent influence on the reproductive system and fruit development.

## 4. Discussion

Here, we present a draft genome of *Eugenia uniflora* (385.1 Mbp), which was used to identify and annotate genes and SSR markers for this species. This genomic resource also facilitated transcriptomic analysis, providing insights into how these populations respond to drought stress.

Genomic analysis identified *Eugenia uniflora*-specific genes and their functional roles, including genes differentially expressed under drought stress, highlighting key biological processes as revealed by COG, KEGG, and CAZy annotations. COG categories highlight the central role of regulatory and stress-responsive pathways. Signal transduction pathways, in particular, are vital for perceiving environmental cues like drought, temperature shifts, and nutrient availability, impacting gene expression, hormone signaling, and defense mechanism [40]. KEGG pathway mapping expanded this functional landscape, revealing 27.16% of the genes are linked to plant-specific metabolic and signaling pathways, with “Biosynthesis of secondary metabolites” being the most enriched. This aligns with previous studies reporting the presence of bioactive compounds—such as flavonoids, tannins, and carotenoids—in leaves and fruits, associated with antioxidant, anti-inflammatory, and nutritional properties [41,42]. CAZy annotation identified genes encoding carbohydrate-active enzymes, mainly from Glycosyl Transferase families GT1 and GT8. GT1 enzymes are involved in glycosylating small molecules (e.g., flavonoids and terpenoids), enhancing their solubility, stability, and bioactivity, especially under stress [43]. GT8 enzymes contribute to the biosynthesis of cell wall polysaccharides like homogalacturonan, which supports cell wall integrity and water retention, key under drought conditions [44]. Notably, GT17 genes were upregulated in Restinga plants, suggesting a role in adaptive metabolism under environmental stress. Together, these findings reveal a coordinated molecular strategy underlying *E. uniflora*’s environmental resilience and emphasize its potential for pharmacological and biotechnological applications.

BUSCO analysis demonstrated that 92.5% of the genes within the *E. uniflora* genome assembly were complete, thereby affirming the high quality of the assembly. The analysis also indicated that 3.5% of genes were absent, which could stem from technical limitations of the BUSCO algorithm or evolutionary events where certain lineages lose highly conserved genes [29]. Furthermore, 4% of genes were fragmented, possibly due to incomplete assembly from short reads, or challenges in locating and predicting genes with complex structures or high divergence [45].

A limitation of the obtained genome is related to the use of short reads that are assembled with de Bruijn graphs and that can collapse heterozygous regions into single contigs. This can lead to an underestimation of the copy number of annotated genes and a reduction in apparent heterozygosity, resulting in inaccurate genetic predictions and a significant statistical over- or under-representation of genic families [46]. This could be solved by incorporating reads from third-generation sequencing technologies (Nanopore and PacBio), which would allow improving the precision in the structural annotation of genes, as demonstrated by the genomes obtained in others Myrtaceae such as *Syzygium malaccense*, *Syzygium aqueum*, *Syzygium jambos*, and *Syzygium syzygioides* [47].

Based on RNA-Seq data from plants of Restinga and Riparian Forest environments, we identified several polymorphic SSR loci between these two contrasting populations. These findings provide a valuable tool for understanding genetic variability and stress responses in this species. A total of 55,019 SSRs were identified, with a huge bias in terms of nucleotide numbers, sequence content, and expression profiles. Nearly 70% of SSRs identified are dinucleotide repeats, of which the AG/CT sequence comprises almost 60% of total SSRs (Fig 10A). A similar profile was reported in another study with *Arabidopsis thaliana* and *Oryza sativa*, where both species’ 5’ UTR gene regions were composed mainly of this same sequence [48]. Although *A. thaliana* did not show the same pattern for untranscribed SSRs, the most prevalent dinucleotide repeat in *O. sativa* was also AG/CT, granted that both species showed a more balanced distribution within dinucleotide repeats. Furthermore, the same study reported a tendency for hexanucleotide repeats in *A. thaliana* to be long strings of A interrupted by another nucleotide, *i.e.,* AAAAAT, indicating a preference for monomeric repeats. This same profile was found in *E. uniflora* and is corroborated by the fact that the most prevalent sequence repeat for each SSR repeat size is variations of (A)_n_S, where S is either C or G (Fig 10B). Yet, to the best of our knowledge, it is not clear how and why this preference for monomeric repeats occurs. A broader study comparing SSR patterns across different kingdoms and phylum identified a shared characteristic of living organisms choosing other dinucleotide repeats over GC/GC, although some clades are an exception [49]. Another tendency observed is that land plants either have AT/AT or AG/CT as their most common dinucleotide repeat, over the previously mentioned GC/GC or AC/GT, which is more prevalent in mammals. This aligns with our data, which shows that *E. uniflora* dinucleotide SSRs are composed mainly of AG/CT and very little GC/GC content. An explanation for the lack of GC/GC repeat content could be that the molecular purification of GC-rich regions by cytosine deamination after methylation, particularly in the context of GC/GC repeats, could lead to increased DNA methylation by the formation of CpG islands.

Expressed SSRs were identified in both *E. uniflora* populations, with a statistically significant increase in the number of exclusive dinucleotide repeats in Restinga populations (Fig 10C), highlighting a potential correlation between SSR patterns and their drought tolerance. This is interesting given the lesser genetic diversity identified in previous studies when comparing plastidial markers [1]. Furthermore, this diversity in SSR sequences has been shown to be related to many developmental processes in *E. uniflora* (Fig 10E), indicating a powerful application of these SSRs as molecular markers to aid phenotype selection and plant breeding. As such, out of the 7,101 expressed SSRs, we successfully recovered 36 candidates with a significant statistical contribution to gene expression levels (p-value < 0.05) (S3 Table). These sequences are either di- or trinucleotide sequences with 5–21 repeats. While most do not present more than one allele, *eug1362:39142* is found to harbor up to five polymorphisms. It would be interesting to evaluate whether these SSRs are themselves the regulatory key in the differential gene expression levels identified, given that they could regulate expression by influencing mRNA function [50], or were vertically inherited together with another genetic mechanism responsible for these alterations. The presence of SSRs within or near genes has been correlated with changes in gene expression in *Arabidopsis* and rice [51], similar to our results for *E. uniflora*. DNA methylation and histone acetylation data from the same study suggest that SSRs may affect gene expression at transcriptional levels by altering local DNA packaging [51]. Moreover, experiments on transcript turnover suggest a posttranscriptional effect, with SSRs enhancing mRNA stability.

Studying abiotic stress in native species like *E. uniflora* is crucial from both ecological and biotechnological perspectives. Abiotic factors can significantly influence plant population dynamics and the evolution of traits that are vital for ecological success, facilitating adaptation to new and diverse environments. As a result, these factors may play a key role in plant diversification and speciation [52]. In this study, two populations of *E. uniflora* (from Restinga and Riparian forest) were compared regarding gene expression regulation under drought conditions. Transcriptomic analysis revealed that the RE population is more responsive to stress than the RF population, exhibiting a higher number of regulated genes. We were interested in exploring the strategies employed by this population to manage water deprivation. Maintaining cell turgor pressure during drought stress is crucial for preventing wilting and supporting growth. Our findings show that Restinga plants employ several mechanisms to protect their cells and reduce water loss. One key strategy is the induction of starch biosynthesis, a well-established process that helps maintain cellular turgor and protect cellular machinery during water scarcity [53,54]. Restinga plants likely achieve this through the induction of the ADP-glucose pyrophosphorylase gene, which encodes a key rate-limiting enzyme in the starch biosynthesis pathway and has previously been implicated in drought tolerance [55]. Additionally, cuticular wax production may represent another important strategy. Cuticular wax forms the first line of defense against environmental stresses and is composed of very long-chain fatty acids (VLCFAs) and their derivatives. The enzyme 3-ketoacyl-CoA synthase (KCS), a key rate-limiting factor in VLCFA biosynthesis, was found to be induced in Restinga plants. Overexpression of *MsKCS10* from alfalfa in transgenic tobacco enhanced drought tolerance by reducing stomatal water loss, improving photosynthesis, and maintaining osmotic potential under drought stress [56].

A common stress response is an increase in cell wall-related proteins. Notably, expansin genes were induced in this plant population. These proteins regulate cell wall extensibility, facilitating cell enlargement and expansion [57]. Expansins play a multifaceted role in drought stress tolerance by improving cell wall flexibility, maintaining water retention, stabilizing cellular structures, and modulating physiological processes such as stomatal regulation and photosynthesis [58].

Glycosylation is a crucial biochemical process that involves the modification of receptor molecules both inside and on the cell surface, playing a key role in maintaining cellular homeostasis and facilitating specific functions. This process is catalyzed by glycosyltransferase family proteins (GTs), with UDP-glycosyltransferases (UDPGTs) being one of the prominent members. Importantly, UDPGTs are induced in Restinga plants and are thought to play a significant role in plant responses to abiotic stresses, particularly drought [59]. Overexpression of *UGT79B2/B3* in *Arabidopsis* enhanced tolerance to cold, drought, and salt stress by regulating anthocyanin accumulation [60]. In tomato, *UDPGT*s can catalyze the glycosylation of ABA and regulate the dynamic equilibrium of ABA levels [61].

Under abiotic stress, the accumulation of reactive oxygen species (ROS) and reactive aldehydes increases are common, primarily due to impaired photosynthesis and respiration. These compounds contribute to oxidative damage. Consequently, the activation of antioxidant and detoxification mechanisms enhances the plant’s ability to tolerate stress. Restinga plants exhibited induction of genes for redox homeostasis, such as Thioredoxin, Early light-induced protein, and iron ascorbate-dependent oxidoreductase, and may employ detoxification mechanisms through Aldo-keto reductase (AKRs) induction, a detoxifying enzyme that reduces toxic aldehydes and ketones that accumulate during abiotic stress [62].

Transcriptional regulation plays a central role in stress responses. Many transcription factors (TFs) exhibit distinct expression patterns in Restinga plants compared to Riparian forest species. Notably, genes encoding for ABI5, WRKY, HD-Zip, GRAS, and bHLH transcription factors show differential regulation, suggesting a unique stress adaptation strategy in Restinga environments. These transcriptional shifts likely contribute to the plant’s ability to withstand the challenging conditions of Restinga habitats.

Restinga plants may also employ epigenetic processes through histone acetylation, methylation, and DNA methylation to regulate gene expression. This is supported by increased levels of pyruvate dehydrogenase, an enzyme essential for producing acetyl-CoA, which provides the acetyl group for histone acetylation, along with the induction of histone acetyltransferases (HATs) [63]. Additionally, histone and DNA methylation are positively regulated in these plants by the induction of histone-lysine methyltransferases (HMTs) and S-adenosylmethionine (SAM) methyltransferases. These epigenetic marks play essential roles in plant response to abiotic stresses [64,65] and likely serve as a “memory system,” helping plants withstand challenges such as drought, high temperatures, and saline soils—conditions commonly found in *Restinga* environments. These marks may enhance Restinga plants’ resilience by enabling rapid and adaptive responses to environmental changes.

Nutrient uptake is typically reduced under water deprivation [66]. However, this population appears to maintain ion homeostasis by upregulating channels that facilitate the uptake of key elements such as sodium, phosphate, magnesium, and iron, as well as regulation of aquaporins, including Major Intrinsic Proteins (MIPs) and Nodulin-like Intrinsic Proteins (NIPs), known for transporting water and small solutes. These mechanisms highlight the remarkable ability of this population to cope with water deprivation.

The association between SSRs and drought-related genes such as those involved in starch biosynthesis, cell wall remodeling, and antioxidant defense mechanisms provides evidence that SSRs may serve as molecular markers for selecting stress-tolerant genotypes. The differential regulation of these SSRs in the Restinga population further suggests that they may be involved in unique strategies that enhance the plant’s ability to cope with water scarcity, thus supporting the adaptive potential of *E. uniflora* in drought-prone environments.

As revealed in this study, molecular markers from different individuals and populations indicate plant genomes exhibit high diversity and a substantial proportion of genomic sequences are not shared among individuals of the same species. However, this study is limited by its reliance on a single sequenced individual of *E. uniflora*, which may not fully represent the species’ genomic diversity across its native range. Given the broad ecological distribution and environmental variability across the species’ habitats, broader sampling across multiple populations is essential to capture the full extent of intraspecific variation. Future research should therefore prioritize expanded sampling and pan-genomic approaches to better understand the evolutionary and ecological dynamics of *E. uniflora*, uncover stress-resilient alleles, and inform conservation and breeding strategies tailored to diverse environmental contexts [67]. Additionally, integrating epigenomic analyses in future studies could provide valuable insights into gene regulation and adaptive plasticity, especially in response to environmental stressors, further complementing genomic findings.

## Supporting information

S1 Fig Venn diagram showing the orthologous gene clusters identified between *E. uniflora* and *E. grandis.* (TIF)

S2 Fig Functional annotation of the *E. uniflora* specific genes using (a) KOG, (b) KEGG and (c) CAZy. (TIF)

S1 Table Annotation of the *E. uniflora* specific genes. (XLSX)

S2 Table Gene ontology of *E. uniflora* specific genes. (XLSX)

S3 Table *E. uniflora* gene ordering based on string interaction. (XLSX)

S4 Table Differentially expressed genes from Restinga population under drought stress. (XLSX)

S5 Table Differentially expressed genes from Riparian Forest population under drought stress. (XLSX)

S6 Table SSRs linked to gene expression. (XLSX)

S7 Table Differential expressed genes with SSR. (XLSX)

S1 File Inclusivity in global research. (DOCX)
