# Supplementary figures and images for: Harnessing genetic diversity: The genomic and transcriptomic insights of Eugenia uniflora for environmental resilience
Source: PLoS One. 2025 Nov 4;20(11):e0333437. doi: 10.1371/journal.pone.0333437 (PMC12585098; doi:10.1371/journal.pone.0333437)

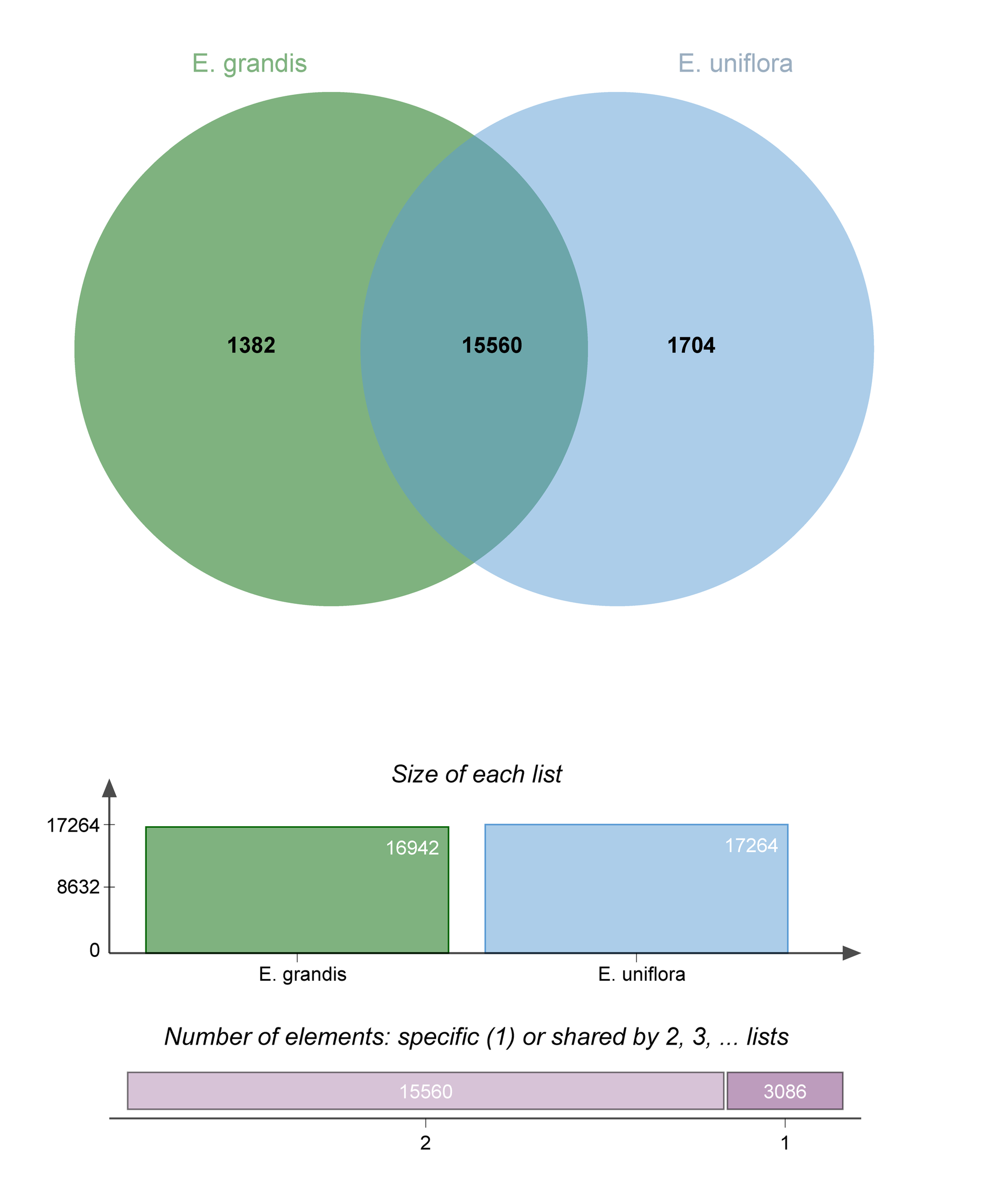

Supplement: S1 Fig — (TIF) [file pone.0333437.s001.tif]

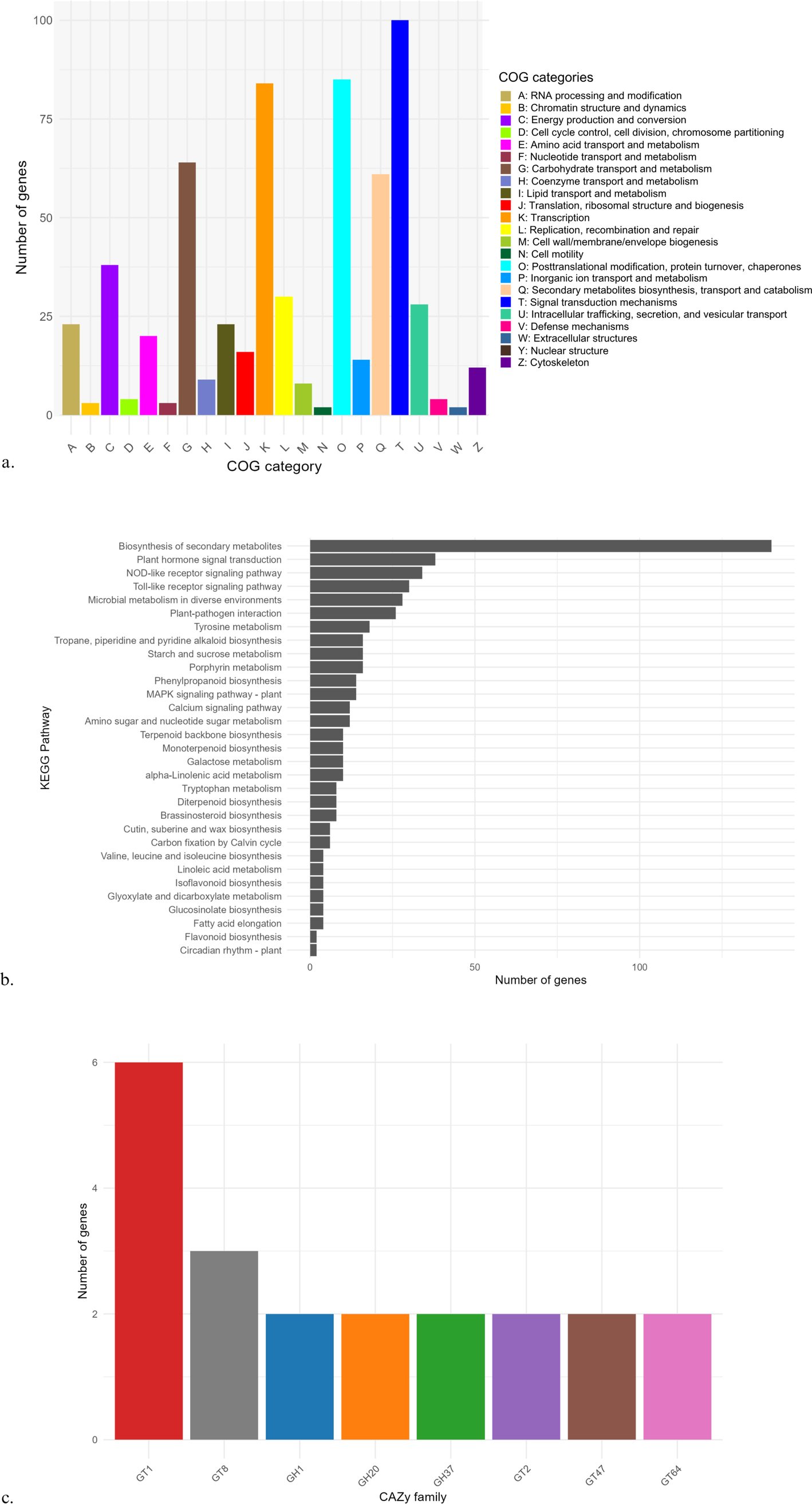

Supplement: S2 Fig — (TIF) [file pone.0333437.s002.tif]
